# A comparison of parametric models for the investigation of the shape of cognitive change in the older population

**DOI:** 10.1186/1471-2377-8-16

**Published:** 2008-05-16

**Authors:** Graciela Muniz Terrera, Fiona Matthews, Carol Brayne

**Affiliations:** 1 MRC Biostatistics Unit, Institute of Public Health, Robinson Way, University Forvie Site, CB2 0SR, Cambridge, UK; 2 Department of Public Health and Primary Care, Institute of Public Health, University of Cambridge, University Forvie Site, CB2 0SR, Cambridge, UK

## Abstract

**Background:**

Cognitive decline is a major threat to well being in later life. Change scores and regression based models have often been used for its investigation. Most methods used to describe cognitive decline assume individuals lose their cognitive abilities at a constant rate with time. The investigation of the parametric curve that best describes the process has been prevented by restrictions imposed by study design limitations and methodological considerations. We propose a comparison of parametric shapes that could be considered to describe the process of cognitive decline in late life.

Attrition plays a key role in the generation of missing observations in longitudinal studies of older persons. As ignoring missing observations will produce biased results and previous studies point to the important effect of the last observed cognitive score on the probability of dropout, we propose modelling both mechanisms jointly to account for these two considerations in the model likelihood.

**Methods:**

Data from four interview waves of a population based longitudinal study of the older population, the Cambridge City over 75 Cohort Study were used. Within a selection model process, latent growth models combined with a logistic regression model for the missing data mechanism were fitted. To illustrate advantages of the model proposed, a sensitivity analysis of the missing data assumptions was conducted.

**Results:**

Results showed that a quadratic curve describes cognitive decline best. Significant heterogeneity between individuals about mean curve parameters was identified. At all interviews, MMSE scores before dropout were significantly lower than those who remained in the study. Individuals with good functional ability were found to be less likely to dropout, as were women and younger persons in later stages of the study.

**Conclusion:**

The combination of a latent growth model with a model for the missing data has permitted to make use of all available data and quantify the effect of significant predictors of dropout on the dropout and observational processes. Cognitive decline over time in older persons is often modelled as a linear process, though we have presented other parametric curves that may be considered.

## Background

Severe changes in the population pyramid have been acknowledged by international organisations such as the United Nations [1]. It has been estimated that the number of persons older than 60 years will be around 2 billion by the year 2050 [2]. They will represent more than a third of the total population in developed regions. In least developed regions, similar changes in society will still be seen though on a smaller scale [3]. These changes in the population present challenges for society and individuals. Countries need to plan and implement strategies to cope with the increase in the demand of health and social services from the older segments of the population [4].

At the individual level, the loss of physical and mental health are two main threats to the enjoyment of wellbeing in older ages and to the costs and health of carers. Age associated diseases such as dementia including Alzheimer's disease represent serious threats to healthy life of older persons and their carers. These diseases already affect over half a million people in England and Wales [5].

As a central feature of the ageing process, understanding and measuring changes in cognitive function has therefore become a key issue for society and researchers.

Many tests have been used to measure cognition, although the Mini Mental Status Examination [6] is the most widely used tool to assess global cognition. Many population based longitudinal studies of the older population have used this test to both identify individuals who are likely to have dementia cross sectionally and also to investigate decline, despite its origins as a screening instrument for dementia.

The MMSE can detect decline and correlates with biological and neuropathological markers associated with the dementias [7]. It takes integer values in the 0–30 interval, with higher values indicating good cognitive function.

Historically, change has been regarded as an incremental or as a continuous process [8]. When regarded as an incremental process, most authors have calculated change scores to quantify it [9-12] and then grouped individuals according to the magnitude of the change observed. When regarded as a continuous process, various forms of regression analysis, mixed effects models [13], generalised estimating equations [14] and unconditional latent growth analysis [15] have been used to investigate cognitive change.

There is large literature on age-based models fitted to examine change in specific cognitive abilities [16-23] that reported a non-linear decline. However, a review of the literature showed that often [24-28] change in global MMSE has been modelled fitting linear lines without further investigation of the trajectory shape that best describes the process. Yet, evidence suggests that the assumption of MMSE scores changing at a constant rate with time cannot be regarded as satisfactory. For instance, when semi parametric techniques were used to investigate cognitive decline before dementia diagnosis [29], it was found not to be linear. Several other studies have also identified an accelerated loss of cognition before death [30-33]. These findings suggest that further investigation of model shape is needed in order to understand whether rate of decline remains constant with time or not.

Limitations for further exploration of parametric curves that could fit the data were sometimes imposed by the study design. For instance, Backman et al. [24] reflect on the fact that as in the Kungsholmen study only two data waves were available, they could only fit a straight line model as it was the only model identifiable. Yet, they reckon that the issue of investigating other functions deserved further consideration. Korten et al. [27] and Christensen et al. [34] in the Canberra study, were in a similar position due to the lack of a sufficient number of interview waves to assure identification of higher order models.

The effect of attrition is a major issue in longitudinal studies of the older population as individuals are more likely to die during the duration of the study or are too frail to be interviewed. If inferences about population decline are made without accounting for them results are likely to be biased towards less observed decline [35]. Methods previously used to investigate change either use the complete subsample only as in change scores, assume a missing completely at random mechanism without an explicit formulation of a missing data model as in generalised estimating equations [35], or a missing at random mechanism as in the parametric and semiparametric versions of random effects models [35].

In this paper, we fitted a linear, a quadratic and two piecewise latent growth models with the aim of investigating further possible functional shapes to describe cognitive decline in older age. These latent growth models included a model to describe the missing data mechanism and were parameterised within the selection models framework defined by Little and Rubin where the joint distribution of the complete data and a model for the missing data are factorised, conditional on the complete data model [35]. Latent growth models not only permit the estimation of latent variables that quantify marginal change but also individual's growth factors are estimated. Using them, it is possible to calculate model implied person specific trajectories and therefore, satisfy the need of investigating change at the individual level. Moreover, latent growth models allow for the latent variables to be regressed on relevant covariates known to be informative of the process of interest. Latent growth models are robust against a missing at random mechanism even so, the formulation of an explicit model for the missing data mechanism has several advantages. First, as theoretical considerations point to the last observed cognitive score as a strong predictor of dropout, the missing data model allows us to account for it within the model likelihood. Second, it permits the investigation of predictors of dropout at each time point of the study. Additionally, it allows for the possibility of conducting a sensitivity analysis under a range of possible assumptions about the dependence of dropout on unobserved scores [36].

All analyses were carried out in Mplus, Version 4 [36]. This software package uses maximum likelihood estimation techniques. The models fitted required numerical integration for the computations performed using Monte Carlo techniques.

## Methods

### The data

Data from the Cambridge City over 75 Cohort Study were used to perform the analysis proposed. The main aims of the study were to establish the incidence and prevalence of dementia. Initially, people aged at least 75 years old in 1985 who were registered at five primary care practices in the Cambridge City area were invited to participate in the Cambridge City over 75 Cohort study [37], then called Hughes Hall Project for Later Life. Those who agreed (95% response rate) were screened by trained interviewers in their homes at which socio- demographic information was collected.

This screening interview was followed by a more detailed clinical interview of all participants suspected of having dementia, identified by a cut point of 23 on the MMSE, and a third of those with scores of 24 and 25 points on the MMSE.

Further waves of interviews were carried out to establish incidence of dementia [38]. After baseline (T0), interviews were carried out an average of 2 (T2), 7 (T7) and 9 (T9) years later. Data from the first four interviews were examined here.

Characteristics of individuals at baseline are shown in Table 1.

**Table 1:** Baseline characteristics of the sample analysed.

Variable	N (%)
Non manual profession	755 (38%)
Left school aged <= 14 years old	1582 (70%)
Female	1266 (63%)
Walks unaided around town or block	1582 (73%)
Married	773 (33%)
Mean ± st. dev. age at baseline	81 ± 5 yrs.
Mean ± st. dev. MMSE (median)	
Baseline	24.5 ± 5.2 (26)
T2	23.0 ± 7.2 (25)
T7	24.1 ± 5.3 (25)
T9	26.3 ± 4.5 (25)

A small number of participants did not take part in some study waves but rejoined the study later. Data from them were only considered up to their first dropout to produce a non intermittent data set. All participants were observed at baseline, but 43.4, 24.5 and 16.6% of the sample dropped out for the first time at T2, T7 and T9 respectively. These include drop out due to death.

### Analysis

MMSE scores from the first four interview waves of the study were analysed using latent growth analysis where the latent variables (mean curve parameters) were adjusted for risk factors for cognitive decline such as education, gender and age cohort at baseline.

**Figure 1:**
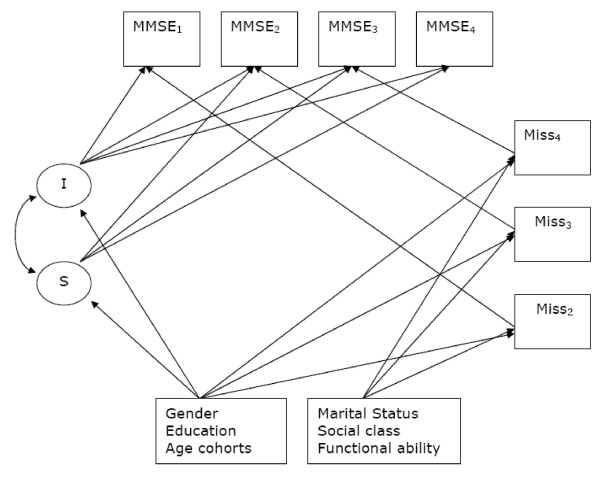
Diagram representing the latent growth linear model fitted.

Four models were fitted: a linear, a quadratic and two piecewise models with change points at T2 and T7 respectively. The parameterisation of the models was such that the models' mean intercept represent mean cognitive status at baseline and the mean slope, average rate of change in cognition between T0 and T2. In the quadratic model, the quadratic term represents the rate of change in the linear term. In the piecewise models, the slope of the second piece represents the change in the linear term of the first piece after the change point. Correlations between latent variables were estimated unless fixed to zero to assure model fit. Figure 1 shows a diagram of the linear model fitted with equations in Appendix 1. Latent variables are represented within ellipses and observed variables such as MMSE scores, missing data indicators and risk factors for cognitive decline that were used to adjust the latent variables and the missing data model are represented within rectangles. Straight arrows show how variables are related whereas curved ones represent correlations between the latent variables.

A missing at random mechanism [35] was fitted using a simplified Diggle and Kenward's model [39]. That is, a logistic regression was fitted to model the probability of an individual missing an interview with the individual's previous score, education, gender, marital status, functional ability, social class and age cohort at baseline as covariates.

Numerical integration is needed to estimate maximum likelihood parameters in latent growth curve models with random effects and missing data. As standard fit indices are not appropriate for these types of models, in order to identify the model that best fits the data, a Bayesian Information Criteria (BIC) based selection was undertaken. As the BIC is a measure of fit that considers the likelihood with a penalisation for the number of parameters, the most parsimonious model would be the one with the lowest BIC and therefore, that model should be chosen as the one with best fit.

#### Sensitivity of models to normality assumption

Latent growth models require normality of residuals. On visual exploration of this assumption, some departure of normality was identified. To improve the normality of the data, MMSE scores were transformed using the transformation *Y*_*it *_= log(31-*MMSE*_*it*_) previously applied in the literature [5] and models ran again. Some improvement in model fit was achieved. The quadratic model was the model with the lowest BIC value, and hence, the one with best fit in the transformed scale.

**Figure 2:**
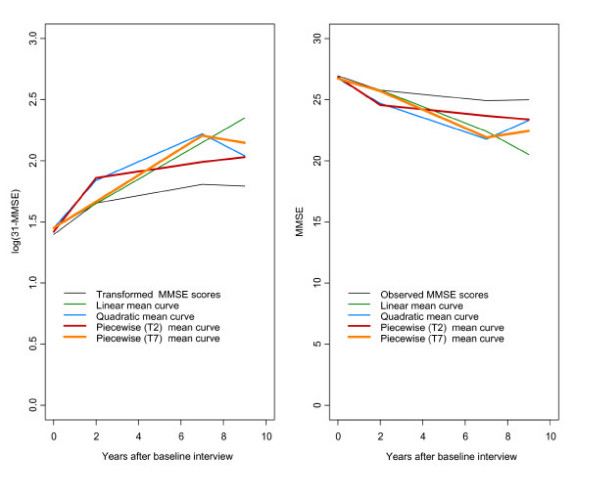
The plot on the left shows the mean curve of scores obtained after applying the transformation *Y*_*it *_= log(31-MMSE_*it*_) to MMSE scores and the mean estimated curves obtained from fitting a linear, a quadratic and two change point models to these transformed scores. On the right, the back-transformed curves are shown.

Figure 2 shows estimated mean curves obtained from fitting the models to transformed scores and the curves obtained after back- transforming these estimated curves.

The results obtained from fitting the models to raw data are presented furtehr for two main reasons. First, the main aim of our investigation was to show a range of parametric models that can be used to model cognitive change taking into account the missing data. And second, the interpretation of the back transformed models is complex when non-linear or non-isometric transformations are applied in these models.

## Results

Table 2 shows model parameters of the four models fitted. For the four models, parameters presented consist of the estimated mean initial score represented by the intercepts of the mean curves and rate of change represented by the slopes of the models. For the quadratic term, an estimate of the curvature of the mean curve was also estimated; whilst for the piecewise models, there is an estimate of the slope of the second piece.

**Table 2:** Mean estimates and standard errors of latent variables of growth models fitted

Model with explicit formulation of missing data model				
Parameter	Linear	Quadratic	Piecewise with change point at T2	Piecewise with change point at T7

Intercept	25.4 (0.2)*	25.4 (0.2)*	25.4 (0.3)*	25.3 (0.3)*
Slope1	-0.9 (0.1)*	-1.14 (0.2)	-1.13 (0.2)	-0.8 (0.1)
Quadratic term	-	0.07 (0.02)	-	-
Slope2	-		-0.2 (0.1)	0.4 (0.4)

* significant residual variance

In the linear and quadratic models, there was significant variability about the models' intercept and slope, an indication of heterogeneity of individuals about these parameters. The correlation between the intercept and slope in the linear model was estimated as 0.64 (st. error = 0.08), and in the quadratic, at -0.47 (1.79). The correlation of the quadratic term with the intercept was estimated at -0.11 (st. error = 0.07) in the quadratic model, and with the slope at -0.03 (st. error = 0.02). In the two piecewise models the variances of the two slopes were fixed at zero to achieve better model fit.

At all interviews, MMSE scores before dropout of those who dropped out were significantly lower than for those who remained in the study (t-tests, df = 1957, level 5%, p < 0.01 for all interviews).

All models consistently showed those with lower previous scores and lower functional ability as more likely to miss an interview. At the second and third interviews, women were less likely to dropout. Those in the youngest cohort interview were also found to be more likely to be observed in the fourth interview.

BIC values of the four models fitted were estimated at 27956.1, 27890.1, 28217.2 and 28179.3 for the linear, quadratic and the piecewise models with change points at T2 and T7 respectively. These BIC values identified the quadratic model as the model with best fit.

The same models were run without an explicit formulation of a model for the missing data. In all models, estimated slopes indicated less decline. For instance, in the case of the linear model, the mean slope was estimated at -0.7 (st. error = 0.09), a value that suggests a smoother decline when compared to the slope estimated by the linear model formulated within the selection modelling framework (estimate = -0.9, st error = 0.1). Factor scores were also estimated at different values, in particular, individual specific slopes were consistently estimated at higher values than the slopes estimated by the model with a formultation of the missing data model.

One of the main advantages of our proposed models is that they can be easily extended to model a non-ignorable missing data mechanism. This could be used to perform a sensitivity analysis of the missing data assumptions. We extended the work of Dufouil et al. [40], where a sensitivity analysis of the missing data assumptions was conducted by choosing a range of plausible values for the regression coefficients of the unobserved scores on the logistic regression used to model the missing data mechanism. In Dufouil et al. [40], a constant value was considered over time as the assumed coefficient for the unobserved MMSE score. In our example, under the hypothesis that the unobserved MMSE score has an increasing effect on the probability of the individuals missing an interview over time, we considered increasing coefficients for these unobserved scores.

To illustrate the method, results obtained from the quadratic model are presented in Tables 3 &4. These results show, for instance, that the non-ignorable model produced larger odds of missing an interview given the last observed score than the missing at random missing data model.

**Table 3:** Odds ratio (95% C.I.) missing data model results.

Interview			
Risk Factor	Second	Third	Fourth

Previous MMSE	0.82 (0.79–0.85)	0.81 (0.78–0.84)	0.79 (0.80–0.85)
Education	0.85 (0.64–1.13)	0.99 (0.75–1.54)	0.84 (0.55–1.27)
Marital status	0.84 (0.65–1.08)	0.82 (0.59–1.54)	0.84 (0.56–1.24)
Social class	0.93 (0.72–1.20)	0.90 (0.63–1.27)	1.04 (0.70–1.55)
Functional ability	0.52 (0.38–0.71)	0.43 (0.26–0.68)	0.30 (0.19–0.62)
Gender	0.88 (0.69–1.10)	0.60 (0.46–0.86)	0.51 (0.33–0.77)
Younger cohort	0.73 (0.54–1.01)	0.43 (0.27–0.71)	0.27 (0.13–0.56)
Medium cohort	0.84 (0.61–1.14)	0.58 (0.35–0.94)	0.47 (0.22–1.05)

**Table 4:** Odds ratio (95% C.I.) non-ignorable missing data model results.

Interview			
Risk Factor	Second (assumed MMSE_2 _= -0.10)	Third (assumed MMSE_7 _= -0.15)	Fourth (assumed MMSE_9 _= -0.20)

Unobserved MMSE	0.90	0.86	0.81
Previous MMSE	0.87 (0.84–0.90)	0.90 (0.87–0.94)	0.84 (0.81–0.88)
Education	0.79 (0.62–0.99)	0.85 (0.66–1.10)	0.61 (0.39–0.96)
Marital status	0.83 (0.67–1.03)	0.81 (0.63–1.04)	0.77 (0.52–1.13)
Social class	0.89 (0.72–1.11)	0.82 (0.64–1.05)	0.88 (0.59–1.30)
Functional ability	0.55 (0.41–0.72)	0.46 (0.30–0.70)	0.34 (0.15–0.76)
Gender	0.79 (0.64–0.98)	0.55 (0.43–0.72)	0.51 (0.33–0.79)
Younger cohort	0.88 (0.66–1.18)	0.65 (0.43–0.98)	0.62 (0.26–1.48)
Medium cohort	0.94 (0.70–1.27)	0.72 (0.47–1.16)	0.93 (0.38–2.31)

## Discussion

Overall, we propose that the use of latent growth models to investigate cognitive decline at the marginal and individual level provides researchers with good opportunities that represent advancement with respect to methods previously used in the field. Change scores are known to be severely affected by reliability issues [41]. Furthermore, covariates that might be informative about the cognitive process cannot be included in most versions of change scores.

On the other hand, some regression based analysis are not suitable for the correlated measures that are observed in longitudinal data. Despite generalised estimating equations being more flexible in this regard, estimates of change at the individual level are not easily obtained. When mixed effects models are used, random effects are often not reported [42].

Latent growth analysis is flexible enough to estimate the full trajectory of change at the mean level also producing individual's growth factors that permit the reconstruction of individual's trajectories that are of interest to clinicians [43]. Distributional assumptions of latent growth models require normality of residuals.

To improve the fulfilment of this assumption, we transformed scores and ran all models using the resulting scores. Results obtained from transformed data showed some improvement in the satisfaction of the normality assumption and identified the quadratic as the model that fits the data best in the transformed scale. However, thoughtful consideration needs to be exercised in the interpretation of back-transformed results as parameter estimates apply to the transformed scale not the original MMSE scale. As the purpose of the paper is the illustration of the method proposed, and the interpretation of results is natural in the original MMSE scale, we opted for the presentation of all other results in the MMSE scale.

Our analysis of cognitive decline in older persons over a 9 year longitudinal has identified the quadratic curve as the one that best fits the data. The quadratic model suggests an initial drop followed by a deceleration in the rate of decline expressed by a positive quadratic term. A relatively small annualised rate of decline was estimated (around 1.14 points) in our study, a finding that agrees with other studies. For instance, Jacqmin-Gadda [26] found very small change after the first two interviews, in which an improvement in MMSE scores was detected. Similarly, Feskens [44] and Brayne [45] working with two waves of data have also identified a small change in the samples analysed. Also, although working with an expanded MMSE, researchers in the Canadian Study [11] found that 70.6% of the sample did not change between the first two interviews, another 62.5% exhibited little change between interviews two and three and almost 50% remained stable for the 10 years of the study. A similar non-linear decline has also been identified in studies where change in specific cognitive abilities [16-23] was examined. This behaviour may be a consequence of a survivor effect or could also be explained by the plateau described by Wernicke and Reischies [46], after which individuals do not decline any further.

Attrition has severe effects on studies of older people. In the literature different levels of analysis of the missing data mechanism present in the samples can be found. For instance, in studies where change scores were used to model change [47-50] individuals with missing observations were removed from the analysis sample. The main consequence of such practice is a bias in results towards less observed decline [35]. In Jacqmin-Gadda et al., Amieva et al., Korten et al. and Royall et al. [26,27,29,51] where mixed random effect models and latent growth models were fitted, estimates that are robust against a missing at random mechanism were obtained but an explicit model for the missing data mechanism was not provided. Dufouil et al. [40] investigated marginal change accounting for the missing observations using inverse probability weighting to reduce the bias produced on observed data assuming an informative missing data mechanism. All other studies have relied on the robustness of the estimates against a missing at random mechanism.

Instead, we have opted for the actual modelling of the mechanism using Diggle and Kenward's logistic regression model. This was important as by explicitly modelling the missing data, we were able to quantify the effect of the last observed score on probability of dropout at each interview and to fully account for the effect of previous MMSE scores within the full model likelihood. We believe that the explicit formulation of a model for the missing data mechanism is a novel practise that is necessary to fully inform the measurement model in order to provide an accurate description of the cognitive change experimented by older individuals.

Furthermore, it raises further opportunities for conducting sensitivity analyses of the models with respect to the missing data as shown.

Our results agree with previous studies in which a missing at random mechanism was present in the data. Yet, we found that women are less likely to drop out than men at later interviews, as opposed to the findings by Van Beijsterveldt et al. [52]. Our findings regarding older individuals being more likely to drop out agree with theirs.

Perhaps one of the disadvantages of our study is the large amount of missing data after the second interview. This is common in most studies of the older population and the fact that a missing data model was implemented should smooth its consequences regarding parameter estimates. Our models could benefit from the inclusion of further variables such as the person's cardiovascular disease history, genetic data and social network, for instance. They would not only inform the measurement model, but also the missing data model. Another key variable that would benefit the understanding of the process is the reason for dropout. If information was available about the cause of drop out, then it would be plausible to perform a competing risk analysis or a multiple group analysis discriminating by reason for drop out. This is under investigation at the moment.

Another possible limitation of the study is the fact that we considered a growth model based on 'time in study' rather than on the individual's age at each interview, an approach that facilitated the fit of the missing data model based on fixed times of observation. The discussion about the most suitable temporal matrix to describe cognitive change is contentious [53-56] and when multiple cohorts are examined, age-based models are likely to provide a better description of age related cognitive decline. However, by adjusting our models by age defined cohorts, we compensated the impact of using a fixed time basis and were able to model the missing data process simultaneously.

Our research has proved that making parametric assumptions when investigating a process as complex as the loss of cognitive function as measured by the MMSE in older ages deserves thorough consideration.

## Conclusion

Our study has showed that cognitive decline in older persons is not a linear process. The quadratic growth model, identified as the one with best fit, permitted the estimation of initial mean cognitive status, rate of decline and of its change. Estimates of person specific parameters were produced.

Significant heterogeneity between individuals in initial cognitive status and rate of decline was identified.

By fitting a latent growth model, we maximised the use of all data available to investigate cognitive change in older persons. Individuals with lower previous cognitive scores have been identified as more likely to drop out.

## Competing interests

The authors declare that they have no competing interests.

## Authors' contributions

GM ran the analysis and produced the manuscript, FM contributed with comments on analysis and manuscript and CB contributed with comments on the manuscript. CB is a principal investigator of the CC75C study. All authors read and approved the final manuscript.

## Appendix 1

The latent linear growth model represented in Figure 1 can be formulated in mathematical terms as:

*MMSE*_*it *_= *λ*_0*t*_*I*_*i *_+ *λ*_1*t*_*S*_*i *_+ *e*_*it*_

*I*_*i *_= α_00 _+ α_01 _*Z*_*i *_+ *u*_0*i*_

*S*_*i *_= α_10 _+ α_11 _*Z*_*i *_+ *u*_1*i*_

where *i *= 1...*N*, with *N *the number of individuals in the sample and *t *= 0,2,7,9 the number of years past after the initial interview.

λ_0*t *_and λ_1*t *_are factor loadings with λ_0*t *_fixed to one, and *e*_*it*_; *u*_0*i *_and *u*_1*i *_normally distributed random errors such that *E*(*e*_*it*_) = 0 for *i *= 1...*N*, *t *= 0,2,7,9; *E*(*u*_0*i*_) = 0 for *i *= 1...*N*, *E*(*u*_1*i*_) = 0 for *i *= 1...*N*;*Cov*(*e*_*it*_,*u*_0*i*_) = 0 and *Cov*(*e*_*it*_,*u*_1*i*_) = 0 for *i *= 1...*N*, *t*= 0,2,7,9; *Cov*(*u*_1*j*_,*u*_1*i*_) = 0 and *Cov*(*u*_0*j*_,*u*_0*i*_) = 0 for *i *≠ *j*.

The missing data mechanism was modelled using a logistic regression model, where the probability of individual *i *being missing at time *t*, *p*_*it *_was modelled as:

log it(*p*_*it*_) = *γ*_*it*_*MMSE*_*i*(*t*-1) _+ Σδ_*i*ν*i*1 _+ ε_1τ_;

where ν_*i*1 _are model covariates and ε_1τ _are residuals that follow a logistic distribution.

## Appendix 2

Basic Mplus code for linear model:

TITLE:

DATA: (data file location)

VARIABLE: NAMES ARE (name declaration);

CLASSES = c(1);

USEVARIABLES = sc1 sc2 sc3 sc4 married sclass mob

educ miss2 miss3 miss4 women young interm;

MISSING = ALL(-999);

CATEGORICAL ARE miss2 miss3 miss4;

ANALYSIS: TYPE = MIXTURE MISSING RANDOM;

algorithm = integration;

integration = montecarlo;

MODEL:

%OVERALL%

i s | sc1@0 sc2@2 sc3@7 sc4@9;

i s ON educ women young interm;

miss2 ON sc1 educ married sclass mob women young interm;

miss3 On sc2 educ married sclass mob women young interm;

miss4 On sc3 educ married sclass mob women young interm;

sc3; sc2;

%c#1%

i s ON educ women young interm;

OUTPUT: cinterval;

SAVEDATA:

RESULTS ARE (file location);

SAVE = FSCORES;

## Pre-publication history

The pre-publication history for this paper can be accessed here:


